# Assessing Porous Media Permeability in Non-Darcy Flow: A Re-Evaluation Based on the Forchheimer Equation

**DOI:** 10.3390/ma13112535

**Published:** 2020-06-03

**Authors:** Simon Tupin, Makoto Ohta

**Affiliations:** Biomedical Flow Dynamics Laboratory, Institute of Fluid Science, Tohoku University, Sendai 980-8577, Japan; makoto.ohta@tohoku.ac.jp

**Keywords:** permeability, non-Darcy flow, Forchheimer equation, TPMS, tissue engineering scaffolds

## Abstract

In a recent paper published in Materials (Castro et al., 2019), the permeability evaluation of triple periodic minimum surface samples was carried out experimentally. Darcy’s law was used under unsuitable conditions, resulting in an underestimation of the results. In this comment, we highlight the problem and propose a new estimation of the permeability using the Forchheimer equation, which is better suited to the experimental conditions.

## 1. Introduction

Triple Periodic Minimum Surfaces (TPMS) are mathematically defined geometries that are of great interest for the development of tissue engineering scaffolds. This method makes it easy to create homogeneous porous structures with controlled porosity and stiffness. In a recent paper, Castro et al. [1] study the influence of the unit cell design on the macroscopic permeability of TPMS. Perfusion experiments were performed on 3D printed replicas to assess the water permeability of each structure. Unfortunately, the theory chosen for this evaluation does not suit the experimental conditions.

## 2. Theory

Darcy’s law is the fundamental law governing fluid flow through porous media. It is a proportional relationship between the flow rate of the fluid through the porous medium and the pressure gradient in the direction of the flow:

(1) $$-\frac{\partial P}{\partial x}=\frac{\mu Q}{kA}=\frac{\mu }{k}v$$

where *P* is the pressure, *x* is the direction of the fluid flow, μ is the fluid dynamic viscosity, *Q* is the flow rate, *k* is the permeability, *A* is the cross-sectional area and *v* is the superficial velocity. Permeability is here a constant that does not depend on the velocity.

Darcy’s law is an approximation that is valid only in a limited range of low velocities (Re<10) [2]. In order to describe higher velocity flows (i.e., non-Darcy flows), Forchheimer [3] added in Equation (1) a second term representing kinetic energy due to inertial effects:

(2) $$-\frac{\partial P}{\partial x}=\frac{\mu }{k}v+\beta \rho v^{2}$$

where β is the Forchheimer coefficient and ρ is the fluid density.

## 3. Permeability Re-Evaluation and Discussion

**Figure 1:**
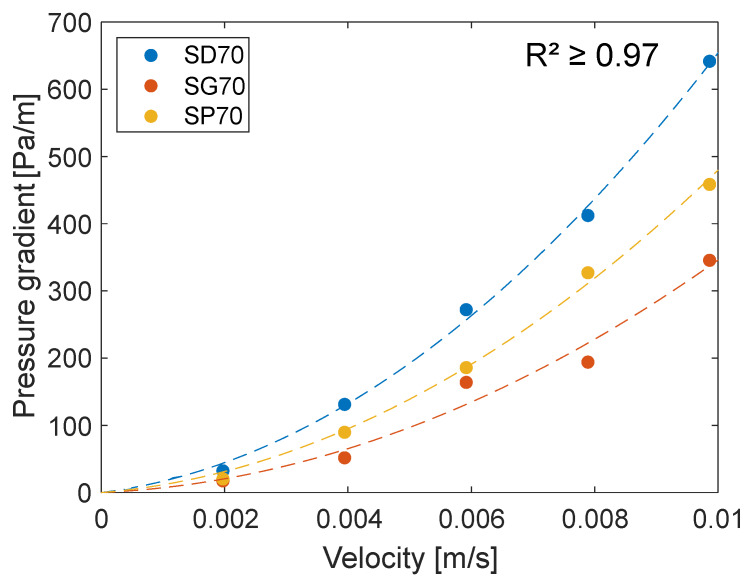
Original data, extracted from Figure 4 in [1] using WebPlotDigitizer [4], and corresponding fits (dashed lines) using the Forchheimer Equation (2).

In the commented article, perfusion experiments were performed in a range of flow rates corresponding to non-Darcy flow conditions (29<Re<145). The reported permeabilities, evaluated with Darcy’s law, differed as the flow rate changes (Figure 4 in [1]), demonstrating the invalidity of Equation (1) in this study. In order to better estimate the permeability of the TPMS samples, the original data were processed using the Forchheimer Equation (2) (Figure 1).

Results revealed that unit cell geometry affects the permeability and the Forchheimer coefficient (Table 1). As porosity was the same in all samples, other microstructure parameters are responsible for these differences and need to be studied. In a similar study [5], the specific surface area of TPMS samples was found to be inversely proportional to permeability, as defined in the Kozeny–Carman equation. Porous media can also be anisotropic, exhibiting different permeabilities when tested in different directions [6,7].

Although the classification of the samples is similar to that reported in the commented paper, permeability values differ. The use of Darcy’s law under invalid conditions induced an underestimation of permeability, as Forchheimer’s term becomes important. Permeability differences were −36±14,−67±1,−79±6,−81±3 and −85±3%, respectively, for flow rates of 20, 40, 60, 80 and 100 mL/min.

The non-Darcy effect, *E*, defined as the ratio between the pressure gradient consumed by liquid-solid interactions (Forchheimer’s term) and the total pressure gradient [8], makes it possible to quantify this phenomenon and to select better experimental conditions:

(3) $$E=\frac{\beta \rho v^{2}}{-\partial P/\partial x}$$

**Figure 2:**
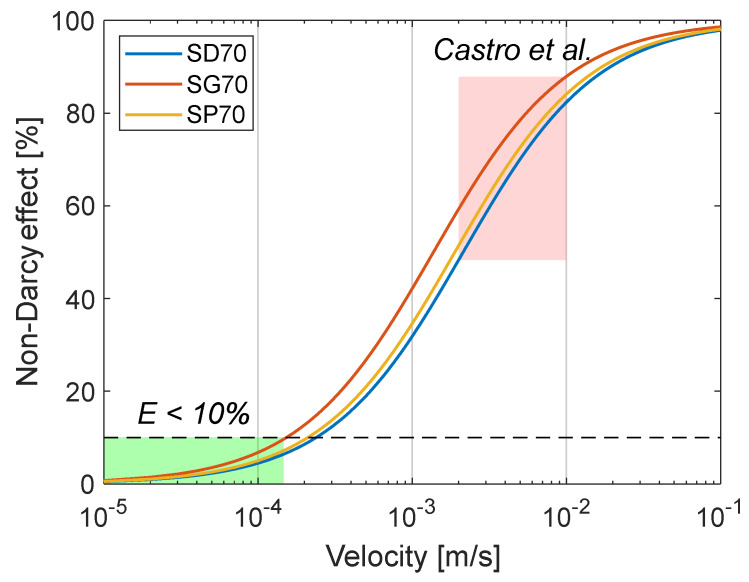
Evolution of the non-Darcy effect, E, estimated for the three TPMS samples. The red and green areas represent respectively the experimental conditions selected in [1] and the velocity range to minimize non-Darcy effects.

Figure 2 shows the evolution of the non-Darcy effect of the studied samples over a wide range of velocities. In the experiments conducted in the original paper, the non-Darcy effect is large (48%<E<88%), leading to an underestimation of the permeability (26% to 88 %). The ideal superficial velocity to minimize the non-Darcy effect (i.e., E<10%) would be ≤0.15 mm/s (i.e., ≤1.52 mL/min). This is consistent with the boundary conditions typically chosen for the study of tissue engineering scaffolds [9].

## 4. Conclusions

The choice of boundary conditions for scaffold permeability experiments must be made carefully so as not to underestimate the result. In the case of non-Darcy flow (i.e., Re≫1), a theory that takes into account inertial effects, such as the Forchheimer equation, should be preferred.

## Figures and Tables

**Table 1:** Permeability, *k*, and Forchheimer coefficient, β, evaluated on the TPMS samples using the Forchheimer Equation (2).

Sample	*k* [m${}^{2}$]	β [m${}^{-1}$]
SD70	$8.679\times 10^{-8}$	5390
SG70	$2.397\times 10^{-7}$	3044
SP70	$1.314\times 10^{-7}$	4035
